# Adipose tissue content of alpha-linolenic acid and the risk of ischemic stroke and ischemic stroke subtypes: A Danish case-cohort study

**DOI:** 10.1371/journal.pone.0198927

**Published:** 2018-06-11

**Authors:** Christian Sørensen Bork, Stine Krogh Venø, Søren Lundbye-Christensen, Marianne Uhre Jakobsen, Anne Tjønneland, Philip C. Calder, Kim Overvad, Erik Berg Schmidt

**Affiliations:** 1 Department of Cardiology, Aalborg University Hospital, Aalborg, Denmark; 2 Human Development & Health Academic Unit, Faculty of Medicine, University of Southampton, MP887 Southampton General Hospital, Southampton, United Kingdom; 3 Department of Clinical Medicine, Aalborg University, Aalborg, Denmark; 4 Unit of Clinical Biostatistics, Aalborg University Hospital, Aalborg, Denmark; 5 Atrial Fibrillation Study Group, Aalborg University Hospital, Aalborg, Denmark; 6 National Food Institute, Technical University of Denmark, Kgs. Lyngby, Denmark; 7 Danish Cancer Society Research Center, Copenhagen, Denmark; 8 National Institute for Health Research Southampton Biomedical Research Centre, University Hospital Southampton NHS Foundation Trust and University of Southampton, Southampton, United Kingdom; 9 Department of Public Health, Section for Epidemiology, Aarhus University, Aarhus, Denmark; University of Illinois, UNITED STATES

## Abstract

**Background:**

The plant-derived omega-3 fatty acid alpha-linolenic acid (ALA) may reduce the risk of cardiovascular disease.

**Objective:**

We have investigated associations between the content of ALA in adipose tissue and the risk of ischemic stroke and its subtypes.

**Methods:**

Incident cases of ischemic stroke among participants enrolled into the Danish Diet, Cancer and Health cohort (n = 57,053) were identified by linkage with the Danish National Patient Register. Subsequently, all potential cases were validated and classified into ischemic stroke subtypes. The fatty acid composition of adipose tissue was determined by gas chromatography in cases and in a randomly drawn sub-cohort (n = 3500). Statistical analyses were performed using weighted Cox regression.

**Results:**

During a median of 13.4 years of follow-up, 1735 cases of total ischemic stroke were identified including 297 cases of large artery atherosclerosis, 772 cases of small-vessel occlusion, 99 cases of cardio-embolism, 91 cases with stroke of other etiology and 476 cases with stroke of undetermined etiology. The median content of ALA in adipose tissue within the sub-cohort was 0.84% (95% central range: 0.53–1.19%). Multivariable analyses showed a U-shaped association between adipose tissue content of ALA and the rate of total ischemic stroke, but this association was not statistically significant (*p* = 0.172). In analyses of ischemic stroke subtypes, we observed a statistically significant U-shaped association between ALA and the rate of ischemic stroke due to large artery atherosclerosis (*p* = 0.017), whereas no appreciable association was observed between ALA and the rate of small-vessel occlusion (*p* = 0.427). A positive but statistically non-significant association was observed between ALA and the rate of ischemic stroke due to cardio-embolism (*p* = 0.162).

**Conclusions:**

The content of ALA in adipose tissue was statistically non-significantly U-shaped associated with risk of total ischemic stroke. For ischemic stroke subtypes a statistically significant, U-shaped association with large artery atherosclerosis was observed.

## Introduction

Alpha-linolenic acid (ALA) is an essential fatty acid which is found mainly in plant oils, seeds and walnuts, but it can also be found in varying concentrations in other foods such as green leafy vegetables, whole grain-cereals, margarines, mayonnaises, potatoes, dairy-products and meat [1–3].

ALA has been associated with several beneficial effects important for development of cardiovascular disease including reduced vascular inflammation [4,5], impaired platelet aggregability [6] and a reduced atherosclerotic plaque burden [7,8]. However, controversy remains whether ALA is associated with a lower risk of ischemic stroke.

ALA has been suggested to be an important nutrient, possibly explaining the protective effect on coronary heart disease (CHD) provided by a Mediterranean diet [9]. However, while some observational studies [2,10–13] have suggested that ALA intake may be associated with a lower risk of CHD, other observational studies have not confirmed these findings [1,14–18]. Previous observational studies investigating the association between ALA intake and the risk of ischemic stroke are sparse and have given inconsistent results [14–16,19,20]. One explanation may be that most previous studies did not distinguish between subtypes of ischemic stroke. This is important because ALA could play different roles in ischemic stroke subtypes due to differences in underlying etiologies. In addition, studies based on dietary intake may be prone to measurement error due to self-reported food intakes and inaccurate food composition tables which may lead to underestimation of potential associations. Furthermore, ALA intake may be difficult to quantify in food questionnaires because sources such a plant oils and margarines often are included in convenience foods [21]. In contrast, biomarker studies may provide objective measures of ALA exposure, and adipose tissue is considered the best biomarker of long-term intake of many fatty acids including ALA [22,23].

The objective of this study was to investigate the associations between the content of ALA in adipose tissue and the risk of ischemic stroke and ischemic stroke subtypes. We hypothesized that adipose tissue content of ALA would be inversely associated with development of ischemic stroke and ischemic stroke subtypes.

## Patients and methods

### Study population and study design

This case-cohort study was based on data from the Diet, Cancer and Health cohort, which previously has been described in detail [24]. Briefly, the cohort was established between 1993 and 1997 by inviting 160,725 men and women to participate. All eligible participants were native Danish citizens without a previous diagnosis of cancer, aged 50–64 years, and living in the urban areas of Copenhagen and Aarhus. Potential participants were retrieved through the Danish Civil Registration System [24]. A sub-cohort of 3500 participants was drawn randomly from the cohort.

We excluded participants with a diagnosis of cancer before baseline that was not yet registered in the Danish Cancer Registry at the time of invitation. Also, participants registered with a diagnosis of stroke before enrollment as well as participants for whom information was missing on exposure or other covariates were excluded.

The study complied with the Declaration of Helsinki. The Diet, Cancer and Health cohort has been approved by the Health Research Ethics, Capital Region of Denmark, and the Danish Data Protection Agency. All participants gave written informed consent at inclusion [24].

### Measurement of adipose tissue content of ALA

An biopsy of subcutaneous adipose tissue from the buttock was taken at baseline from all participants using a Luer-lock system (Terumo, Terumo Corp, Tokyo, Japan) consisting of a needle, a venoject multi-sample Luer adaptor and an evacuated blood tube, according to the method of Beynen and Katan [25]. Samples were stored in liquid nitrogen vapour until analysis. The adipose tissue content of ALA was quantified among all cases and the sub-cohort participants with the use of gas chromatography at a specialized lipid laboratory as described previously [1,26]. Before analysis, the biopsies were thawed and tissue was removed to a glass and prewarmed at 50 ^o^C for ten min and subsequently dissolved in heptane at 50 ^o^C and transesterified by 2mol/L potassium hydroxide in methanol at 50 ^o^C for two min according to the IUPAC standard methods for analysis of oils, fats and derivates. The fatty acid composition was measured using a Varian 3900 gas chromatograph with a CP-8400 auto sampler (Varian, Middleburg, The Netherlands) equipped with a flame ionization detector. Split injecting mode, a CP-sil 88 60m x 0.25mm capillary column and temperature programming (90 ^o^C to 210 ^o^C) were used. Helium was used as carrier gas. Peak retention times and area percentages of fatty acids were identified using commercially available standards (Nu-check-Prep, Inc., US) [26]. Adipose tissue content of ALA was expressed as the weight percentage of total fatty acids. The inter-assay coefficient of variation for the assessment of ALA in adipose tissue was 1.9%.

### Identification of cases

Incident cases of ischemic stroke were identified through the Danish National Patient Register that was established in 1977 and includes information on discharge diagnoses from all hospitals in Denmark [27].

Potential stroke cases included participants registered with either a primary or secondary discharge diagnosis of stroke according to the International Classification of Diseases (ICD) (ICD-8: 430, 431, 433, 434, 436.01, or 436.90 and ICD-10: I60, I61, I63 or I64). Stroke was defined as a disease with rapid onset of focal or global neurologic deficit of vascular origin persisting beyond 24 hours or leading to death. However, patients with focal neurological deficits of shorter duration were also considered as stroke cases if CT/MR imaging showed a recent stroke [28]. All potential stroke cases were validated by a physician with neurological experience and classified according to the Trial of Org 10172 in Acute Stroke Treatment (TOAST)-classification [29] based on assumed etiology [28]. The TOAST-classification separates cerebral infarctions into five groups: large artery atherosclerosis, small-vessel occlusion, cardio-embolism, stroke of other etiology and stroke of undetermined etiology based on clinical findings, brain imaging, imaging of extra cranial arteries, laboratory tests, electrocardiograms, and echocardiography.

Participants were followed from baseline until the first registration of stroke, death, emigration, or end of follow-up in November 2009.

### Covariates

At baseline, participants completed a detailed questionnaire on health status, social factors and lifestyle such as length of schooling, smoking habits, physical activity, history of hypercholesterolemia and/or use of lipid-lowering medication, history of hypertension and/or use of anti-hypertensive medication and a history of diabetes mellitus [24]. Information on alcohol intake and diet was obtained from a validated 192-item semi-quantitative food frequency questionnaire filled in at baseline [30,31]. The questionnaires were checked for reading errors and missing information by technicians who also performed anthropometric measurements including height, weight and waist circumference of the participants [24].

Information on a history of atrial fibrillation/flutter before baseline was obtained by record linkage with the National Patient Register (ICD-8: 42793, 42794 & ICD-10: I48). All potential ischemic stroke risk factors were selected a priori to data analysis.

### Statistical analyses

The associations between adipose tissue content of ALA and the rate of ischemic stroke and ischemic stroke subtypes were investigated using hazard ratios (HRs). HRs and 95% confidence intervals (CIs) were calculated using weighted Cox proportional hazard regression allowing for separate baseline hazards between men and women and with age as underlying time axis. All cases were assigned a weight equal to one, whereas all non-cases in the sub-cohort were assigned a weight calculated as the ratio between the number of non-cases in the cohort after exclusions divided by the number of non-cases in the sub-cohort [32]. Individual weights were calculated for cases of total ischemic stroke and for each ischemic stroke subtype. A robust variance estimator was used for estimating standard errors. This weighting scheme has been shown to perform well in a simulation study [33].

The adipose tissue content of ALA was included as a continuous variable using restricted cubic splines with three knots. The knots were placed at the 10th, 50th and 90th percentile as recommended by Harrell [34]. Splines were plotted to visually assess the shape of the associations with the median as reference and the spline curves were formally tested against a horizontal line using Wald tests. In further analyses, the adipose tissue content of ALA was included as a categorical variable in quintiles with the lowest quintile as reference.

We examined the association between adipose tissue content of ALA and the risk of ischemic stroke in three different models. In model 1A baseline age (years, continuous) was included in order to ensure comparison of participants for whom the exposure was of the same age. In model 1B, baseline information on the following established ischemic stroke risk factors was added: length of schooling (≤7, 8–10, or >10 years), smoking (never, former, current 1–14, 15–24, or ≥24 g tobacco/d), physical activity (inactive, moderately inactive, moderately active, or active), waist circumference adjusted for body mass index (cm, continuous) and alcohol intake (g/d, continuous). In model 2, we adjusted for all the covariates of model 1B and in addition for the following clinical characteristics, which may both be considered potential confounders and potential intermediate variables: self-reported history of hypercholesterolemia or use of lipid-lowering medication (yes, no, or unknown), self-reported history of hypertension or use of anti-hypertensive medication (yes, no, or unknown), self-reported history of diabetes mellitus (yes, no, or unknown), and a diagnosis of atrial fibrillation/flutter recorded in the Danish National Patient Register (yes, no). Adjustments for continuous variables were performed using restricted cubic splines with three knots.

The spline plots were shown for the 95% central range of adipose tissue content of ALA and presented with 95% confidence bonds. In sensitivity analyses, we plotted the whole exposure range of adipose tissue content of ALA and modified the number and placement of the knots.

The proportional hazard assumption was evaluated by plotting scaled Schoenfeld residuals.

Potential differences in the underlying dietary pattern related to adipose tissue content of ALA were assessed graphically in a radar plot comparing the median intake of selected foods and beverages among participants in the highest and lowest quintile of adipose tissue content of ALA.

Data were analyzed using Stata statistical software (version 14; StataCorp LP), and a p-value <0.05 was considered statistically significant.

## Results

A total of 57,053 men and women accepted to participate in the Diet, Cancer and Health cohort study. We excluded 2355 participants because they either had a diagnosis of cancer (n = 569) or stroke (n = 597) before entry, or had missing baseline information on the primary exposure (n = 350) or covariates (n = 961).

During a median of 13.4 years (95% central range: 3.9–15.2) of follow-up, 1735 validated cases of total ischemic stroke with complete information on covariates were identified (S1 Fig). The incidence rate of total ischemic stroke was 2.46 cases per 1000 person years. Total ischemic stroke cases included 297 cases of large artery atherosclerosis, 772 cases of small-vessel occlusion, 99 cases of cardio-embolism, 91 cases with stroke of other etiology and 476 cases with stroke of undetermined etiology. The incidence rate was 0.42 per 1000 person years for large artery atherosclerosis, 1.10 per 1000 person years for small-vessel occlusion and 0.14 per 1000 person years for cardio-embolism.

Baseline characteristics of the participants in the sub-cohort and among total ischemic stroke cases are shown in Table 1, while baseline characteristics among cases of ischemic stroke subtypes are given in S1 Table. Known ischemic stroke risk factors were more prevalent among participants who became cases compared with participants within the sub-cohort. Accordingly, we observed a larger proportion of men and participants with higher age, a shorter duration of schooling, larger waist circumference and a higher alcohol intake and a larger proportion of physically inactive and current smokers among cases. A history of hypercholesterolemia, hypertension and diabetes mellitus was also more prevalent in subsequent cases. Atrial fibrillation/flutter was more prevalent among cases of total ischemic stroke and cases classified with cardio-embolism, ischemic stroke of other etiology and stroke of undetermined etiology compared with the sub-cohort.

**Table 1:** Baseline characteristics among cases and sub-cohort participants.

		Sub-cohort (n = 3185)	Total ischemic stroke cases (n = 1735)
Gender, %			
	Males	54.1	61.8
	Females	45.9	38.2
Age at enrollment, years		56.3	58.8
Duration of schooling, %			
	≤7 years	32.7	40.7
	8–10 years	45.0	42.7
	>10 years	22.3	16.6
Smoking, %			
	Never	34.8	24.6
	Former	29.3	25.8
	Current <15 g/d	13.5	15.5
	Current 15–25 g/d	15.7	23.8
	Current ≥25 g/d	6.8	10.3
Physical activity, %			
	Inactive	11.0	14.7
	Moderately inactive	30.4	30.2
	Moderately active	23.7	21.4
	Active	35.0	33.7
Waist circumference, cm ^a^^,^ ^b^		91.1 (73.9, 104.5)	93.6 (74.9, 105.8)
Alcohol intake, g/d^a^		13.9 (0.2, 85.0)	14.5 (0.0, 93.6)
Clinical characteristics, %			
	Hypercholesterolemia	7.8	10.7
	Hypertension	15.6	28.4
	Diabetes mellitus	2.0	4.2
	Atrial fibrillation/flutter	0.9	1.4

^a^ Median; 2.5th–97.5th percentiles in parentheses

^b^ Adjusted for body mass index

### Association between ALA and ischemic stroke and ischemic stroke subtypes

The median content of ALA in adipose tissue within the sub-cohort was 0.84% (95% central range: 0.53–1.19%).

**Fig 1:**
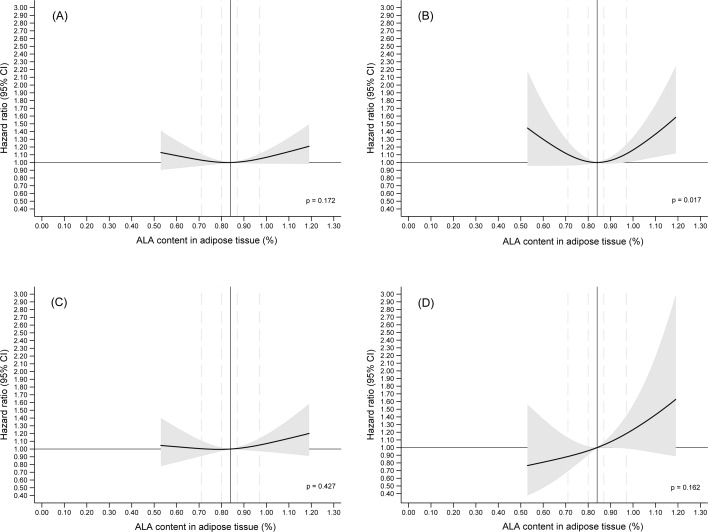
The content of alpha-linolenic acid in adipose tissue and the risk of incident total ischemic stroke and ischemic stroke subtypes. The multivariable analyses were adjusted for established risk factors (model 1B) with median adipose tissue content as reference (solid vertical line). The 20th, 40th, 60th, and 80th percentiles of adipose tissue content of ALA are marked by dashed lines. Only 2.5th–97.5th percentiles of ALA are shown.

Multivariable analyses of adipose tissue content of ALA modeled as a restricted cubic spline and adjusted for established ischemic stroke risk factors (model 1B) showed a U-shaped association between ALA in adipose tissue and the rate of total ischemic stroke, but this association was not statistically significantly different from a horizontal line (*p* = 0.172) (Fig 1). In multivariable analyses (model 1B) of ischemic stroke subtypes, we observed a U-shaped association between adipose tissue content of ALA and the rate of large artery atherosclerosis and this association was statistically significantly different from a horizontal line (*p* = 0.017) (Fig 1). In analyses of the association between ALA in adipose tissue and the rate of small-vessel occlusion, we observed a weak positive association above the median ALA content, however this was not statistically significant (*p* = 0.427). Analyses of the association between ALA in adipose tissue and rate of cardio-embolism showed a positive association, which was not statistically significant (*p* = 0.162) (Fig 1). Analyses of associations between adipose tissue content of ALA and the rate of ischemic stroke of other etiology and rate of ischemic stroke of undetermined etiology are given in S2 Fig.

Analyses of the adipose tissue content of ALA in quintiles and risk of ischemic stroke and ischemic stroke subtypes are shown in Table 2 and S2 Table.

**Table 2:** Quintiles of adipose tissue content of ALA and hazard ratios for total ischemic stroke and ischemic stroke subtypes.

		Cases	Model 1A^a^		Model 1B^b^		Model 2^c^	
		(n)	HR	95% CI	HR	95% CI	HR	95% CI
Total ischemic stroke								
	0.31–0.71%	361	1 (reference)		1 (reference)		1 (reference)	
	0.71–0.80%	338	0.94	0.77, 1.13	0.95	0.78, 1.16	0.93	0.76, 1.15
	0.80–0.87%	295	0.80	0.66, 0.98	0.86	0.70, 1.06	0.89	0.72, 1.09
	0.87–0.97%	357	0.93	0.77, 1.12	0.93	0.76, 1.14	0.92	0.75, 1.13
	0.97–1.69%	384	1.02	0.85, 1.24	1.01	0.82, 1.23	1.03	0.84, 1.27
Large artery atherosclerosis								
	0.31–0.71%	67	1 (reference)		1 (reference)		1 (reference)	
	0.71–0.80%	50	0.75	0.51, 1.11	0.72	0.48, 1.08	0.72	0.48, 1.08
	0.80–0.87%	43	0.63	0.42, 0.94	0.63	0.41, 0.96	0.66	0.43, 1.01
	0.87–0.97%	64	0.91	0.63, 1.30	0.83	0.56, 1.22	0.85	0.58, 1.26
	0.97–1.69%	73	1.07	0.75, 1.53	0.95	0.65, 1.40	0.99	0.68, 1.46
Small-vessel occlusion								
	0.31–0.71%	158	1 (reference)		1 (reference)		1 (reference)	
	0.71–0.80%	150	0.96	0.75, 1.24	1.00	0.77, 1.31	0.98	0.75, 1.28
	0.80–0.87%	138	0.86	0.66, 1.11	0.96	0.73, 1.25	0.98	0.74, 1.28
	0.87–0.97%	162	0.97	0.76, 1.25	1.02	0.78, 1.33	1.01	0.77, 1.33
	0.97–1.69%	164	1.02	0.79, 1.30	1.05	0.80, 1.38	1.08	0.82, 1.42
Cardio-embolism								
	0.31–0.71%	19	1 (reference)		1 (reference)		1 (reference)	
	0.71–0.80%	17	0.91	0.47, 1.78	1.05	0.53, 2.06	1.04	0.50, 2.14
	0.80–0.87%	18	0.93	0.48, 1.79	1.16	0.59, 2.27	1.18	0.58, 2.42
	0.87–0.97%	15	0.73	0.36, 1.48	0.90	0.43, 1.87	0.87	0.41, 1.85
	0.97–1.69%	30	1.50	0.83, 2.72	1.91	0.98, 3.70	2.02	1.01, 4.03

ALA, alpha-linolenic acid; HR, Hazard ratio; CI, Confidence interval.

Hazard ratios with 95% CI intervals were calculated using weighted Cox regression. All models are adjusted for gender by allowing for separate baseline hazards.

a Model 1A included baseline age

b Model 1B included the variables of model 1A and the following risk factors for ischemic stroke: duration of schooling, smoking, physical activity, waist circumference adjusted for body mass index and alcohol intake.

c Model 2 included the variables of model 1B and the following potential intermediate variables: self-reported history hypercholesterolemia and/or use of lipid-lowering medication, hypertension and/or use of antihypertensive medication, diabetes mellitus, and history of atrial fibrillation/ flutter recorded in the Danish National Patient Register.

In analyses including adjustment for established risk factors (model 1B), a U-shaped pattern of association was observed between quintiles of adipose tissue content of ALA and the risk of total ischemic stroke and large artery atherosclerosis, but the hazards in the second to fifth quintiles were not statistically significantly different from the reference except for the third quintile in analysis of large artery atherosclerosis (HR: 0.63, 95% CI: 0.41–0.96). Additional adjustment for a history of hypercholesterolemia, hypertension, diabetes mellitus and atrial fibrillation/flutter (model 2) also showed a U-shaped pattern of associations, but the individual hazards in second to fifth quintiles were also not statistically significantly different from the reference.

No appreciable or consistent associations were observed between quintiles of adipose tissue content of ALA and the risk of ischemic stroke due to small-vessel occlusion nor the risk of ischemic stroke due to cardio-embolism in either of the models. However, a higher rate of stroke due to cardio-embolism was observed in the highest quintile of adipose tissue content of ALA although this was only statistically significant after adjustment for established ischemic stroke risk factors and potential intermediate variables (model 2).

Sensitivity analyses showed that the models using restricted cubic splines were robust when the location and number of knots were modified. No evidence of deviation from the proportionality assumption was observed in either of the models.

A radar plot of the background diet within the sub-cohort revealed several differences in the median intake of selected foods and beverages among participants in different quintiles of adipose tissue content of ALA (S3 Fig). Participants in the highest quintile of adipose tissue content of ALA had higher intakes of margarines, vegetable oils and mayonnaises, refined cereals, processed meat and fish compared with subjects in the lowest quintile of adipose tissue content of ALA within the sub-cohort, and lower intakes of alcohol, dairy products, peanuts, snacks and fatty potatoes, soft drinks and juices, fruit and vegetables.

## Discussion

In this large case-cohort study, we observed a statistically non-significant U-shaped association between adipose tissue content of ALA and the rate of total ischemic stroke and a statistically significant U-shaped association between adipose tissue content of ALA and the rate of ischemic stroke due to large artery atherosclerosis, whereas no appreciable and no statistically significant association was observed between ALA and the rate of ischemic stroke due to small-vessel occlusion. A positive association was observed between ALA and the risk of ischemic stroke due to cardio-embolism, but this was not statistically significant.

Some strengths and limitations should be mentioned. This study holds the advantage of a prospective design with nearly complete follow-up and case ascertainment in a nationwide register independent of the baseline ALA measurement limiting the potential of selection and information bias. A major strength of this study was the use of adipose tissue samples for determination of ALA exposure, which is considered the gold standard as it may reflect long-term fatty acid intake and metabolism [22]. The use of an objective biomarker also limits the concern of random measurement error. However, repeated measurements of the content of ALA in adipose tissue would have been preferable because changes in dietary habits might have occurred during follow-up. Furthermore, the content of ALA in adipose tissue may be influenced by a combination of several factors such as gender, genetics and background diet [22,23]. We therefore consider the ALA content in adipose tissue a marker of endogenous exposure to ALA. Another major strength of this study was that all cases of ischemic stroke were validated and classified into ischemic stroke subtypes. However, this approach did not allow for gender-specific analyses and overfitting may be a concern in the rare subtypes of ischemic strokes due to a limited number of cases. Therefore, the observed positive association between adipose tissue content of ALA and the risk of ischemic stroke caused by cardio-embolism should be interpreted with caution.

Detailed information on ischemic stroke risk factors was included in the analyses, but residual confounding from known or unknown risk factors may still be of importance for the observed associations. Adjustment for established ischemic stroke risk factors (model 1B) somewhat weakened the observed associations although not consistently. Additional adjustment for a history of hypercholesterolemia, hypertension, diabetes mellitus and atrial fibrillation/flutter (model 2) showed similar patterns of association. However, the interpretation of this model is complicated because these clinical characteristics may represent intermediate steps in the causal pathways between ALA exposure and the risk of ischemic stroke and adjustment for these variables could introduce collider stratification bias. Therefore, we consider model 1B to be the most appropriate model for interpretation. We did not include adjustments for dietary factors in the analyses because the content of fatty acids in adipose tissue was expressed as a percentage of total fatty acids and the content of any individual fatty acids in adipose tissue thus depends on the content of other fatty acids. Furthermore, adjustments for dietary factors would introduce restrictions in the underlying dietary pattern. Therefore, measures of associations including adjustment for dietary factors would have been without a clear interpretation in this study.

To our knowledge, no previous cohort studies have investigated the association between adipose tissue content of ALA and the risk of ischemic stroke or ischemic stroke subtypes. However, few shorter-term biomarker studies have investigated the association between the content of ALA in blood fractions and the risk of ischemic stroke. A case-control study nested within a US cohort of postmenopausal women thus reported a modest inverse statistically non-significant association between serum concentrations of ALA and the risk of total ischemic stroke [35]. A follow-up study from Finland showed a U-shaped pattern of association across quartiles of serum concentrations of ALA and the risk of total ischemic stroke among men [36]. Other previous shorter-term biomarker studies have reported no clear associations between the content of ALA in blood fractions and the risk of ischemic stroke [15,37–40]. A potential explanation for the observed inconsistencies between previous biomarker studies could possibly be differences in the background diet. We used a radar plot to illustrate the content of ALA in adipose tissue as an indicator of the background diet and to evaluate potential confounding from the diet. We observed several differences, but the radar plot did not indicate that a high content of ALA overall reflected a healthy dietary pattern. The intake of ALA in our cohort was derived from a variety of foods with margarines, mayonnaises, whole-grain cereals, butter, potatoes, red meat, vegetable oils, fruit, fatty dairy products and processed meat being the major contributors [1], which might be different from ALA sources elsewhere. In the Nurses’ Health study from the US, the major intake of ALA was also derived from several foods with mayonnaises, oils and vinegar and other salad dressings, margarines, meat, dairy products and green leafy vegetables being the largest contributors [2]. Importantly, the intake of marine omega-3 fatty acids in our study was markedly higher than compared with previous cohort studies that have reported inverse associations between ALA intake and the risk of cardiovascular disease [2,10–13].

This may be important given that a previous study has suggested that ALA in particular may reduce CHD risk when the intake of marine omega-3 fatty acids is low [11].

To our knowledge, no clinical trials have investigated the role of ALA supplementation on the risk of ischemic stroke and limited evidence exists from clinical trials on other cardiovascular outcomes. The Lyon Diet Heart Study reported a significantly lower risk of recurrent myocardial infarction (MI) and cardiac death among participants randomized to a Mediterranean diet high in ALA compared to a prudent Western diet [41,42], but given the nature of the intervention the observed effects could not necessarily be attributed to ALA. Finally, the Alpha-Omega Trial reported a modest statistically non-significant lower risk of major cardiovascular events among participants with prior MI randomized to ALA, compared to marine omega-3 fatty acid supplementation or placebo [43]. It must be emphasized that our study did not evaluate the effect of a Mediterranean diet on ischemic stroke, but a possible association between ALA in adipose tissue and ischemic stroke and subtypes of ischemic stroke. The suggested beneficial effects of a Mediterranean diet on cardiovascular disease may be attributable to the sum of several nutrients rather than a single component.

In conclusion, adipose tissue content of ALA was statistically non-significantly U-shaped associated with risk of total ischemic stroke. For ischemic stroke subtypes a statistically significant, U-shaped association with ischemic stroke due to large artery atherosclerosis was observed.

## Supporting information

S1 Fig Flowchart for selection of sub-cohort participants and cases in the Diet, Cancer and Health cohort. (PDF)

S2 Fig **The content of adipose tissue content of ALA and the risk of stroke of other etiology (A) and stroke of undetermined etiology (B).** The multivariate models are adjusted for ischemic stroke risk factors (model 1B) and presented with the median adipose tissue content of ALA as reference (solid vertical line). The 20th, 40th, 60th, and 80th percentiles of adipose tissue content of ALA are marked by dashed lines. Shaded grey areas show 95% confidence intervals of hazard ratios of ischemic stroke subtypes (curves). Only the 2.5th–97.5th percentiles of ALA are shown.
(PDF)

S3 Fig Radar plot of the median energy-adjusted intake of selected food groups in the highest and lowest quintile of adipose tissue content of ALA. The content of ALA in adipose tissue was indexed according to the overall median intake of the selected food groups (grey solid line) within the sub-cohort (n = 3185). The dots represent percentages-wise differences relative to the overall median intake.
(PDF)

S1 Table Baseline characteristics among cases of ischemic stroke subtypes in the Diet, Cancer and Health cohort. (PDF)

S2 Table Quintiles of adipose tissue content of ALA and hazard ratios for stroke of other etiology and stroke of undetermined etiology. (PDF)
