# Supplementary figures and images for: Adipose tissue content of alpha-linolenic acid and the risk of ischemic stroke and ischemic stroke subtypes: A Danish case-cohort study
Source: PLoS One. 2018 Jun 11;13(6):e0198927. doi: 10.1371/journal.pone.0198927 (PMC5995395; doi:10.1371/journal.pone.0198927)

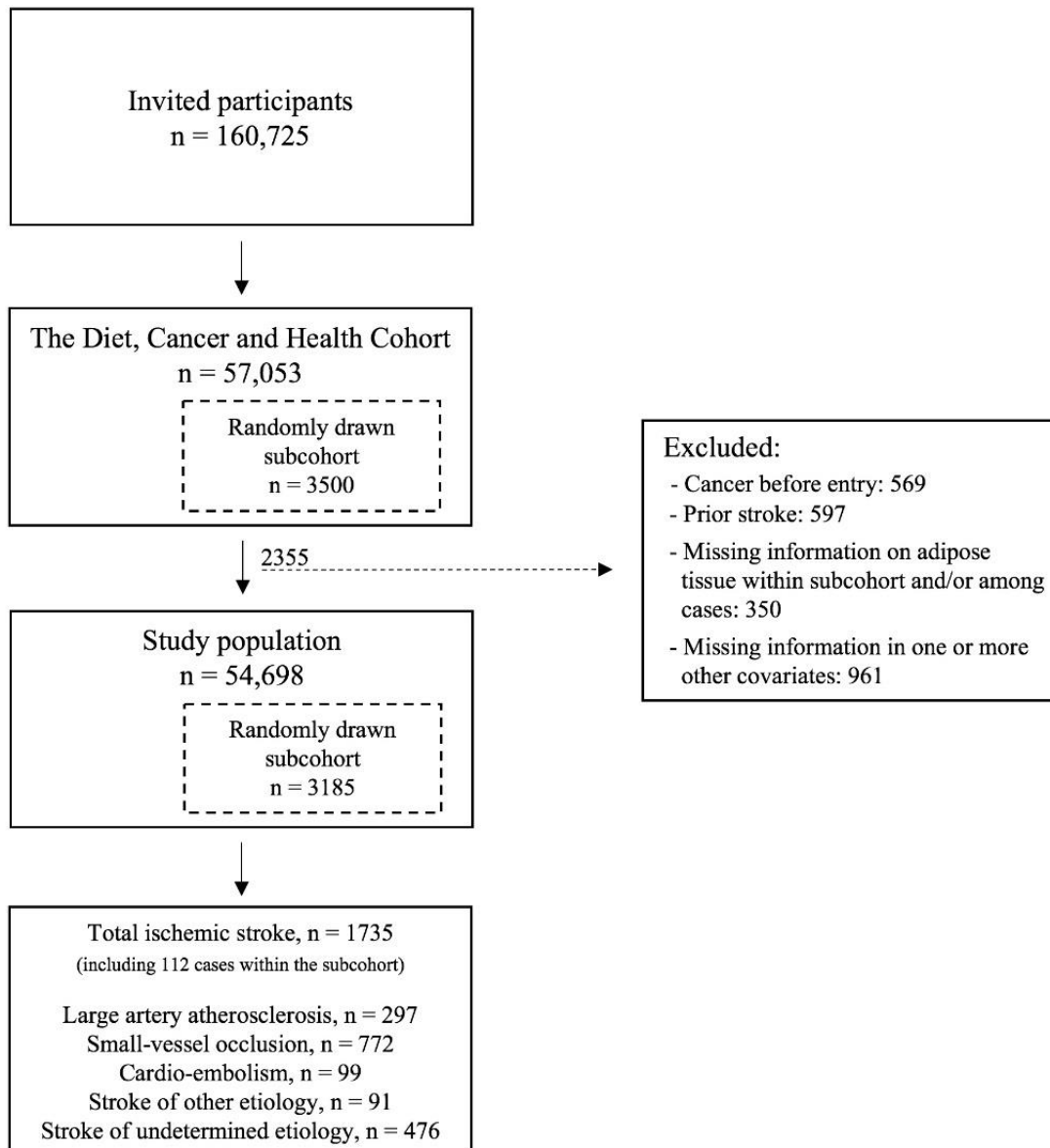

Supplement: S1 Fig — (PDF) [file pone.0198927.s001.pdf]

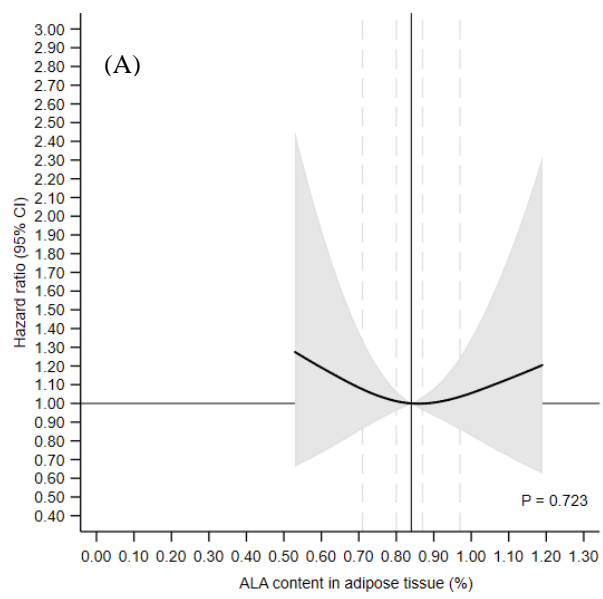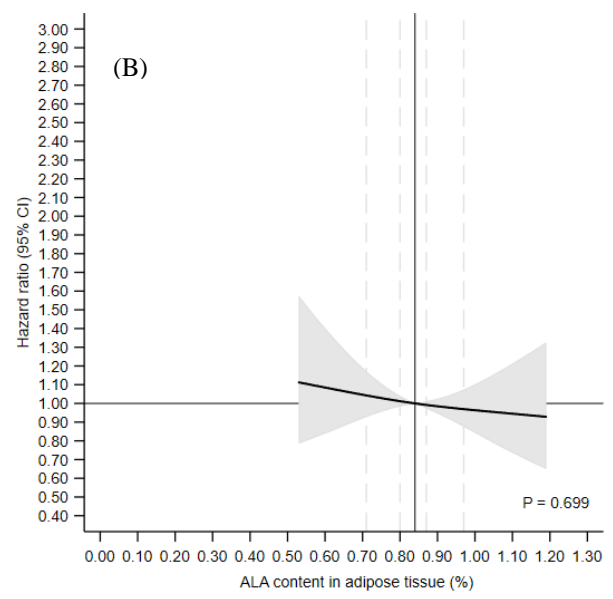

Supplement: S2 Fig — The content of adipose tissue content of ALA and the risk of stroke of other etiology (A) and stroke of undetermined etiology (B). The multivariate models are adjusted for ischemic stroke risk factors (model 1B) and presented with the median adipose tissue content of ALA as reference (solid vertical line). The 20th, 40th, 60th, and 80th percentiles of adipose tissue content of ALA are marked by dashed lines. Shaded grey areas show 95% confidence intervals of hazard ratios of ischemic stroke subtypes (curves). Only the 2.5th–97.5th percentiles of ALA are shown. (PDF) [file pone.0198927.s002.pdf]

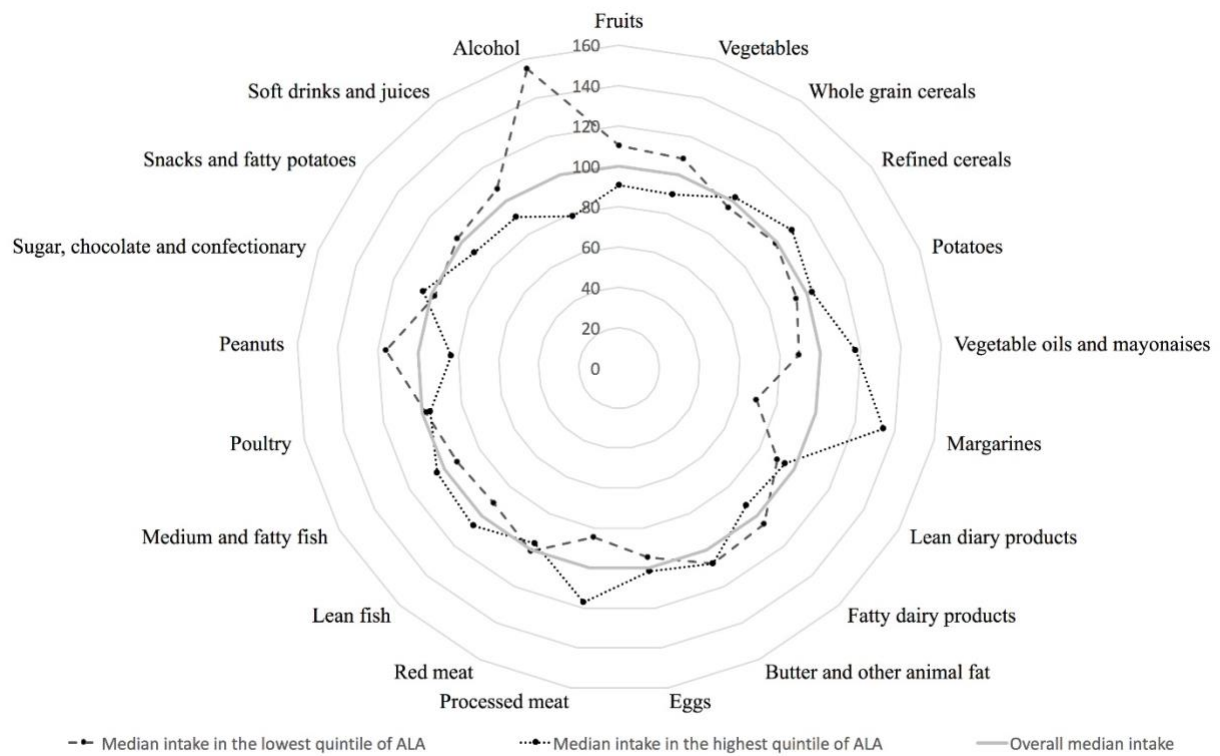

Supplement: S3 Fig — The content of ALA in adipose tissue was indexed according to the overall median intake of the selected food groups (grey solid line) within the sub-cohort (n = 3185). The dots represent percentages-wise differences relative to the overall median intake. (PDF) [file pone.0198927.s003.pdf]
