# Supplementary material for: Adipose tissue content of alpha-linolenic acid and the risk of ischemic stroke and ischemic stroke subtypes: A Danish case-cohort study
Source: PLoS One. 2018 Jun 11;13(6):e0198927. doi: 10.1371/journal.pone.0198927 (PMC5995395; doi:10.1371/journal.pone.0198927)
Supplement: S1 Table — (PDF) [file pone.0198927.s004.pdf]

S1 Table. Baseline characteristics among cases of ischemic stroke subtypes in the Diet, Cancer and Health cohort

	Large artery athero-sclerosis (n = 297)	Small-vessel occlusion (n = 772)	Cardio-embolism (n = 99)	Stroke of other etiology (n = 91)	Stroke of undetermined etiology (n = 476)
Gender, %					
Males	58.6	60.2	67.7	61.5	65.3
Females	41.4	39.8	32.3	38.5	34.7
Age at enrollment, years	58.9	58.5	59.1	56.7	59.5
Duration of schooling, %					
≤7 years	39.1	40.7	40.4	44.0	41.2
8-10 years	46.8	42.6	38.4	40.7	41.6
>10 years	14.1	16.7	21.2	15.4	17.2
Smoking, %					
Never	23.9	27.3	21.2	24.2	21.4
Former	25.3	23.8	30.3	25.3	28.6
Current <15 g/d	13.8	16.1	18.2	16.5	14.7
Current 15-25 g/d	27.3	22.0	21.2	23.1	25.2
Current ≥25 g/d	9.8	10.8	9.1	11.0	10.1
Physical activity, %					
Inactive	13.1	15.7	10.1	13.2	15.3
Moderately inactive	32.0	29.9	31.3	27.5	29.8
Moderately active	25.6	19.0	18.2	20.9	23.5
Active	29.3	35.4	40.4	38.5	31.3
Waist circumference, cm [a, b]	93.2 (75.0, 106.1)	93.1 (75.0, 105.5)	94.3 (72.7, 106.6)	93.7 (75.9, 105.2)	94.6 (73.4, 107.2)
Alcohol intake, g/d[a]	13.3 (0.1, 84.1)	14.9 (0.0, 90.4)	19.0 (0.0, 110.1)	9.4 (0.0, 134.7)	14.0 (0.2, 98.1)
Clinical characteristics, %					
Hypercholesterolemia	8.8	11.1	11.1	8.8	11.6
Hypertension	26.9	27.1	32.3	25.3	31.1
Diabetes	4.4	4.5	3.0	4.4	3.8
Atrial fibrillation or flutter	0.3	0.4	8.1	2.2	2.1

[a] Median and 2.5th - 97.5th percentiles

[b] Adjusted for body mass index
