# Supplementary material for: Adipose tissue content of alpha-linolenic acid and the risk of ischemic stroke and ischemic stroke subtypes: A Danish case-cohort study
Source: PLoS One. 2018 Jun 11;13(6):e0198927. doi: 10.1371/journal.pone.0198927 (PMC5995395; doi:10.1371/journal.pone.0198927)
Supplement: S2 Table — (PDF) [file pone.0198927.s005.pdf]

S2 Table. Quintiles of adipose tissue content of ALA and hazard ratios for stroke of other etiology and stroke of undetermined etiology

	Cases	Model 1A[a]		Model 1B[b]		Model 2[c]	
	(n)	HR	95% CI	HR	95% CI	HR	95% CI
Stroke of other etiology							
0.31-0.71%	14	1 (reference)		1 (reference)		1 (reference)	
0.71-0.80%	25	1.74	0.89, 3.39	1.73	0.90, 3.31	1.74	0.90, 3.36
0.80-0.87%	9	0.63	0.27, 1.46	0.62	0.26, 1.44	0.63	0.27, 1.47
0.87-0.97%	23	1.54	0.78, 3.05	1.40	0.71, 2.73	1.38	0.70, 2.70
0.97-1.69%	20	1.36	0.67, 2.73	1.10	0.56, 2.18	1.14	0.57, 2.29
Stroke of undetermined etiology							
0.31-0.71%	103	1 (reference)		1 (reference)		1 (reference)	
0.71-0.80%	96	0.94	0.69, 1.28	0.95	0.69, 1.31	0.93	0.67, 1.29
0.80-0.87%	87	0.83	0.61, 1.13	0.87	0.63, 1.21	0.90	0.65, 1.26
0.87-0.97%	93	0.85	0.62, 1.16	0.82	0.59, 1.14	0.81	0.58, 1.14
0.97-1.69%	97	0.89	0.66, 1.22	0.85	0.61, 1.19	0.87	0.62, 1.22

ALA, alpha-linolenic acid; HR, Hazard ratio; CI, Confidence interval.
Hazard ratios with 95% CI intervals were calculated using weighted Cox proportional hazard regression. All models are adjusted for gender by allowing for separate baseline hazards.

[a] Model 1A included baseline age

[b] Model 1B included the variables of model 1A and the following risk factors for ischemic stroke: duration of schooling, smoking, physical activity, waist circumference adjusted for body mass index and alcohol intake.

[c] Model 2 included the variables of model 1B and the following potential intermediate variables: self-reported history hypercholesterolemia and/or use of lipid-lowering medication, hypertension and/or use of antihypertensive medication, diabetes mellitus, and history of atrial fibrillation or flutter recorded in the Danish National Patient Register at baseline.
